# Sorting nexin 27 interacts with Fzd7 and mediates Wnt signalling

**DOI:** 10.1042/BSR20150205

**Published:** 2016-02-10

**Authors:** Lixiang Sun, Xiuyong Hu, Wanming Chen, Wei He, Zhiming Zhang, Tuanlao Wang

**Affiliations:** * School of Pharmaceutical Sciences, State Key Laboratory of Cellular Stress Biology, Xiamen University, Xiamen 361102, Fujian, China; † The First Affiliated Hospital of Xiamen University, Xiamen 361003, Fujian, China

**Keywords:** Fzd7, PDZ domain, SNX27, Wnt signalling

## Abstract

SNX27 is the only sorting nexin (SNX) that contains a PDZ domain, which interacts with PDZ-binding motif of target proteins to regulate the trafficking of these proteins. We here showed that SNX27 interacts with Frizzled (Fzd) receptors via PDZ domain interaction. Immunofluorescence microscopy revealed that Fzd7 can be internalized and associate with SNX27-containing endosomal membrane. In addition, SNX27 enhances the endocytosis of Fzd7 and promotes the degradation of Fzd7. Further examination demonstrated that SNX27 inhibits the Wnt regulated transcription activity of TCF/LEF. Our results suggested that SNX27 interacts with Frizzled receptors to regulate the endocytosis and stability of Fzds, and consequently mediates canonical Wnt signalling.

## Abstract (precis)

This work found that sorting nexin 27 (SNX27) interacts with Frizzled receptors (Fzds) through PDZ domain interaction, which act as novel interacting partners for SNX27. Functional investigation of the interaction of SNX27 with Fzd7 revealed that SNX27 promotes the degradation of Fzd7, thus down-regulating Wnt signalling.

## INTRODUCTION

The sorting nexins (SNXs) is a large protein family with over 33 members identified in mammalian system. SNXs is the unique protein family characterized by the presence of Phox homology domain (PX), which binds with phosphatidylinositol phosphates (PtdInsPs), preferentially PtdIns(3)Ps. PX–PtdInsPs interaction directs the recruitment of SNXs to the phospholipid-rich cellular membranes, regulating diverse protein trafficking events, such as endocytosis, protein degradation and recycling [1–3].

SNX27 is unique in SNXs family that contains a postsynaptic density protein-95, discs large, zona occludens 1 homology domain (PDZ), which is responsible for protein–protein interaction by recognizing the PDZ-binding motifs at the carboxyl terminal tail of target proteins [4,5]. SNX27 localizes to the early endosomal membrane through PX domain binding to PtdIns(3)P to regulate surface proteins internalization or sorting from the early endosomes, and the interaction of target proteins with the PDZ domain of SNX27 was proposed the underlying mechanism for this regulation. For examples, SNX27 interacts with 5-HT4αreceptor to direct it to early endosomes [6]. Several investigations indicated that SNX27 regulates the endosomal trafficking and degradation of G protein-gated potassium channels [7–10]. Moreover, SNX27 regulates recycling of β2-adrenoreceptor (β2AR) [5], and the internalization of multidrug resistance-associated protein 4 (MRP4) [11]. Recent findings suggest that SNX27 regulates the trafficking of AMPA-type glutamate receptor (AMPAR) to mediate the synaptic plasticity [12,13]. Physiological investigations demonstrated that functional deficiency in SNX27 is involved in neurological diseases [14–17].

Many proteins regulated by SNX27 belong to the G-protein coupled receptor (GPCR) super family, such as β2AR, 5-HT4R, β1AR and SSTR5 [5,6,18,19]. Frizzled (Fzd) receptor is similar to GPCR with seven transmembrane-spanning domains structurally, and the cytoplasmic domain of Fzd couples to hetero-trimeric G proteins to activate non-cononical and canoni-cal Wnt/Fzd pathway [20–23]. In canonical Wnt signalling pathway, Wnt ligands stimulation triggers β-catenin mediated gene transcription, simultaneously, Fzd will be internalized and undergoes vesicular trafficking pathway [24,25], however, the trafficking of Fzd remains unclear. Amino-acid sequence analysis showed that most of Fzds contain a PDZ-binding motif at the C-terminal tail. However, it has not been reported whether SNX27 interacts with Fzds. In the present study, we found that SNX27 binds to Fzd through PDZ domain interaction, and regulates the canonical Wnt signalling.

## MATERIALS AND METHODS

### Antibodies

The monoclonal antibodies (mAbs) against EEA1 were from BD Biosciences. mAb against Myc-tag (9E10) and mAb against HA tag were obtained from ATCC mAb against GFP and mAb against GAPDH were purchased from Signalway Antibody (SAB). The rabbit polyclonal antibody against HA tag was from Millipore. Rabbit anti-Fzd7 polyclonal antibody was purchased from Abcam. Mouse anti-SNX27 antibody was the gift from Dr Wanjin Hong (Institute of Molecular and Cell Biology, Singapore). HRP-conjugated secondary antibodies were purchased from Pierce. Texas red- or Cy5-conjugated secondary antibodies were from Jackson ImmunoResearch.

### Expression plasmids

The correspondent nucleotide sequences of the C-terminal tail (CT) of Fzd1(627aa-647aa), Fzd6(686aa-706aa) and Fzd7(554aa-574aa) were amplified by PCR from the cDNA of Fzd1, Fzd6 and Fzd7, respectively. The respective PCR products were inserted into the pGEX-4T-1 vector to generate the expression plasmids GST-Fzd1CT, GST-Fzd6CT and GST-Fzd7CT. GST-Fzd1ΔPDZbm and GST-Fzd7ΔPDZbm were generated through similar approaches by deleting the last three amino acids at the C-terminal tail of Fzd1 and Fzd7 to disrupt the PDZ-binding motifs, respectively. For generating HA-Fzd7, the coding region of human cDNA of Fzd7 was cloned into pcDNA3.1(+) vector with a HA tag(YPYDVPDYA) inserted after the signal peptide of Fzd7 protein sequence.

The coding region of human SNX27 cDNA (refer to SNX27a isoform) or SNX27ΔPDZ (with PDZ domain deleted) was cloned into pDmyc vector to construct myc-SNX27 or myc-SNX27ΔPDZ, and recovered by PCR and subcloned into pEGFP-C1 vector to generate GFP-SNX27 and GFP-SNX27ΔPDZ, respectively. myc-SNX27ΔPX (with PX domain deleted) was constructed using similar approaches by subcloning the correspondent coding sequences into pDmyc-vector. All constructs were confirmed by DNA sequencing.

### Cell culture and transfection

Hela cells and MCF7 cells are from ATCC and grow in Dulbecco's modified Eagle's medium (DMEM) (Invitrogen) supplemented with 10% fetal bovine serum (HyClone) in a 5% CO_2_ incubator at 37°C. Transient transfection of plasmids was conducted using TurboFect *in vitro* transfection reagent (Thermo) according to the manufacturer's protocol. G418 (Sigma) was used to generate cells pool stably expressing GFP-SNX27 or shRNA-SNX27.

### Immunofluorescence microscopy

Cells grown on cover glasses were washed with PBSCM (PBS containing 1 mM CaCl_2_ and 1 mM MgCl_2_) and then fixed with 3% paraformaldehyde in PBSCM at 4°C. After sequential washing with PBSCM supplemented with 50 mM NH_4_Cl, cells were permeabilized with 0.1% saponin (Sigma) in PBSCM for 15 min at room temperature, and were subjected for immuno-staining using the primary antibodies followed by Texas-red or Cy5 conjugated secondary antibodies. Immuno-labelled cells or/and GFP-expressing cells were analysed by using confocal immunofluorescence microscope (LSM5 EXITER, Carl Zeiss).

### GST-pulldown, co-immunoprecipitation and western blotting experiments

MCF7 cells expressing the constructs indicated in the text were lysed for 1 h at 4°C in lysis buffer (containing 1% Triton-100, 20 mM Hepes, pH 7.4, 100 mM NaCl, 5 mM MgCl_2_ and EDTA-free proteinase inhibitor cocktail from Roche) and centrifuged at 13000 ***g*** for 30 min at 4°C. For GST-pulldown experiments, the supernatants were incubated with the indicated GST-fusion proteins coupled to the GST-Sepharose 4B resin (GE Healthcare) for overnight. For co-immunoprecipitation, the supernatants were incubated with rabbit-anti-HA tag antibody and protein A agarose (Milipore) for overnight. The beads with bound proteins were resolved by SDS-PAGE after extensively washing and subjected for western blot analysis.

For western blotting, proteins resolved by SDS-PAGE were transferred to nitrocellulose filter and blocked with 5% BSA. After sequentially incubated with the indicated primary antibodies and HRP-conjugated secondary antibodies, the blots were detected by using ECL System (Pierce).

### pSuper-mediated small hairpin RNA interference

Targeting sequence for SNX27 (5'CCAGATGGAACAACGGTTA3') was constructed into pSuper.GFP.neo vector according to the manufacturer's instruction to express shRNA-SNX27. Sequence (5'GATGCAACCACCCACGAAT3') was used for expressing control shRNA-ctrl. The SNX27 knockdown efficiency and the effects on cellular events were examined 72 h after transfection of the shRNA expressing constructs.

### Endocytosis assay

The Hela cells transfected with the HA-tagged Fzd7 and the indicated SNX27 plasmids were starved with FBS-free DMEM for 4 h, then incubated with FBS-free DMEM containing anti-HA antibody (0.1 μg/ml) at 4°C for 1 h. The unbound antibodies were removed by washing with cold PBS for three times. Cells were initiated for internalization of antibodies by adding warm complete DMEM medium containing 100 ng/ml Wnt3a (R&D Systems) and incubated at 37°C. Internalization was stopped with cold PBS at the indicated time. HA-tag antibodies were revealed by Texa-red conjugated secondary antibodies and analysed though confocal immuno-fluorescence microscopy technology. ImageJ software was used for quantitative analysis.

To examine degradation of Fzd7, the cells were triggered for endocytosis with DMEM containing 100 ng/ml Wnt3a and cycloheximide (100 ng/ml) at 37°C. The degradation was monitored by examining the protein level through western blot assay.

### TOPflash assays

TOPflash assay was the common assay for examining Wnt/β-catenin signalling by detecting the transcription activity of TCF/LEF [26]. MCF7 cells expressing GFP-SNX27 or shRNA-SNX27 were co-transfected with pTOPflash reporter plasmids and β-galactosidase plasmids. After 24 h, cells were harves-ted and the luciferase activity was measured using luciferase assay kit (Promega) according to the manufacturer's instruction. The relative luciferase activity was normalized to the β-galactosidase activity.

### Wound healing assay

Wound healing assay was applied to examine the migration capability. Briefly, MCF7 cells expressing GFP-SNX27 or shRNA-SNX27 were grown to 80% of confluence, then the monolayer of cells was wounded with a pipette tip. The cell debris was washed out with PBS. After 48 h, the migration was monitored using an inverted microscope by examining the wound healing.

## RESULTS

### SNX27 interacts with Frizzled receptor via PDZ domain interaction

**Figure 1:**
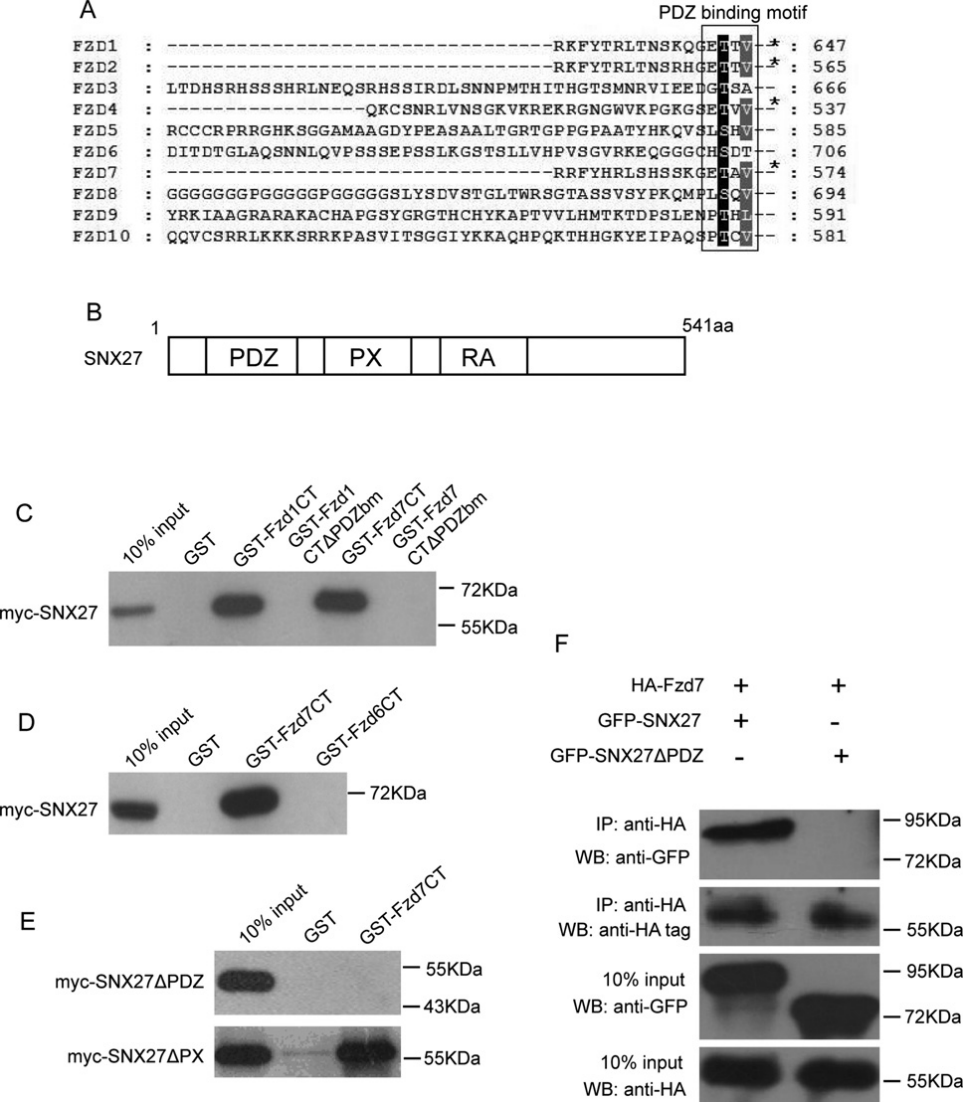
SNX27 interacts with Fzd receptors (**A**) Amino acids sequence alignment analysis showing PDZ-binding motifs at the C-terminal tail of Fzds. (**B**) Schematic diagram showing the structure of SNX27 with PDZ, PX and RA domains. (**C**) GST-pulldown assay showing myc-SNX27 binding to GST-Fzd1CT (C-terminal tail) and GST-Fzd7CT, but not GST-Fzd1CTΔPDZbm (PDZ-binding motif deleted) and GST-Fzd7CTΔPDZbm. (**D**) GST-pulldown assay showing myc-SNX27 interacts with GST-Fzd7CT, but not GST-Fzd6CT. (**E**) GST-pulldown assay showing myc-SNX27ΔPX (PX domain deleted), but not myc-SNX27ΔPDZ can bind to GST-Fzd7CT. (**F**) Co-immunoprecipitation experiments demonstrated that the HA-Fzd7 in full length interacts with GFP-SNX27, but not GFP- NX27ΔPDZ (upper panel).

Frizzled receptors (Fzds) belong to GPCR super protein family, consisting of a group of membrane proteins with N-terminal region flanking outside for binding Wnt ligands, followed by seven trans-membrane domains and a cytoplasmic C-terminal region. The cytoplasmic region is important for interaction with intra-cellular regulator, such as Dishevelled (Dvl) [27,28]. Amino-acid sequences analysis from ten Fzds showed that most of Fzds have a conserved sequence motif “S/T-x-φ” (x indicates any amino acid and φ represents hydrophobic amino acid), except for Fzd3 and Fzd6 (Figure 1A). This motif has been identified as a class I PDZ-binding motif (PDZbm). SNX27 contains three domains, PDZ domain, PX domain and RA domain (Ras-association domain) (Figure 1B). SNX27 is the unique member of SNX family that contains a PDZ domain. The PDZ domain of SNX27 was proved to bind PDZ-binding motif of multiple target proteins. Therefore, SNX27 may potentially interact with Fzds through PDZ domain interaction.

To test the possibilities of the interaction between SNX27 and Fzds, Hela lysates containing expressed myc-SNX27 were subjected for GST-pulldown assay, using fusion protein GST-Fzd1CT (C-terminal tail) or GST-Fzd7CT, which is the GST-fusion protein containing the 21aa of the C-terminal tail of Fzd1 or Fzd7, respectively. As expected, both GST-Fzd1CT and GST-Fzd7CT can bind to SNX27. However, GST-Fzd1CTΔPDZbm or GST-Fzd7CTΔPDZbm (both with last 3 amino acids deleted at the C-terminal tail) doesn't bind to SNX27 (Figure 1C), Interestingly, GST-Fzd6CT (the 21aa at C-terminal tail of Fzd6) doesn't bind to SNX27 either (Figure 1D). The results indicated that the class I PDZ-binding motif at the C-terminal tail of Fzds is crucial for the interaction with SNX27.

On the other hand, we expressed myc-SNX27ΔPDZ (with PDZ domain deleted) or myc-SNX27ΔPX (with PX domain deleted) in Hela cells, respectively, and GST-Fzd7CT was used for GST-pulldown assay to examine whether the PDZ domain of SNX27 is responsible for the interaction, as shown in Figure 1(E), SNX27ΔPDZ will not bind to GST-Fzd7CT, but SNX27ΔPX does, indicating that the PDZ domain, not PX domain, is responsible for SNX27 interacting with the C-terminal tail of Fzds.

To further verify the interaction between SNX27 and Fzd, HA-tagged Fzd7 in full length was co-expressed with GFP-SNX27 or GFP-SNX27ΔPDZ in Hela cells, and the resulted cell lysates were processed for immuno-precipitation experiments. The results demonstrated that SNX27 can be efficiently co-precipitated by Fzd7, but SNX27ΔPDZ cannot. Taken together, our results suggested that SNX27 interacts with Fzd proteins via the PDZ domain of SNX27 binding to the PDZ-binding motif at the C-terminal tail of Fzds.

### SNX27 regulates the endocytosis of Fzd7 receptor

**Figure 2:**
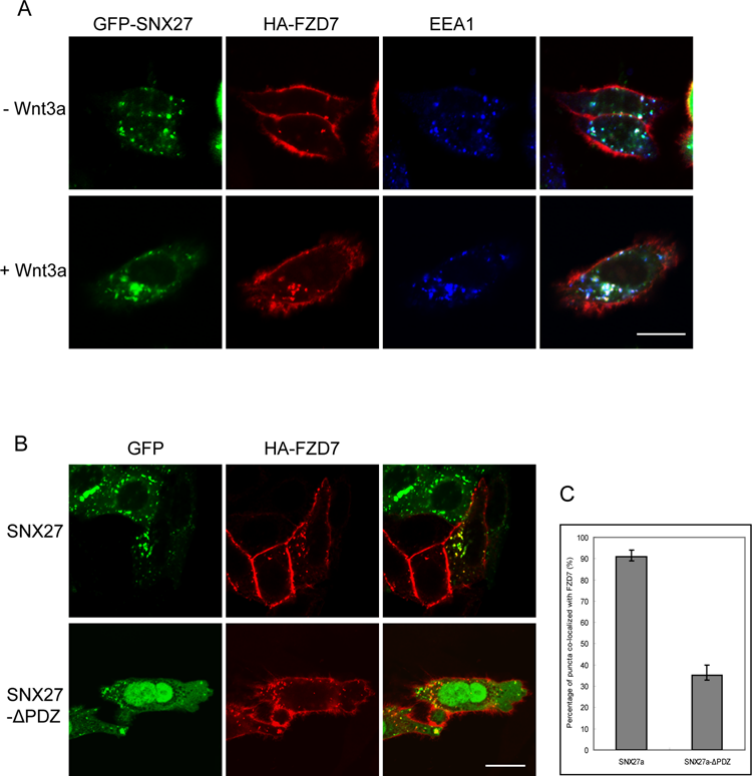
SNX27 regulates the endocytosis of Fzd7 receptor (**A**) Hela cells expressing HA-Fzd7 and GFP-SNX27 were induced for endocytosis with Wnt3a (lower panels) or without Wnt3a (upper panels), showing SNX27 regulates the internalization of HA-Fzd7 from the plasma membrane to the early endosomes, and co-localization of SNX27 with the internalized HA-Fzd7 (lower panels). (**B**) Hela cells co-transfected with HA-Fzd7 with GFP-SNX27 or GFP-SNX27ΔPDZ, then induced for endocytosis. The results revealed that HA-Fzd7 co-localizes with GFP-SNX27 well (upper panels), but the co-localization of HA-Fzd7 with GFP-SNX27ΔPDZ decreases dramatically (lower panels). (**C**) Quantitative analysis from 50 cells showing the co-localization of HA-Fzd7 with GFP-SNX27 or GFP-SNX27ΔPD. Bar=20 μm.

SNX27 locates at the early endosomes to regulate protein endocytosis or recycling. As SNX27 interacts with Fzd7, we next examined the roles of SNX27 in the endocytosis of Fzd7. Hela cells expressing HA-Fzd7 and GFP-SNX27 were incubated with HA-tag antibody, which will bind to HA-tag at the extracellular region of HA-Fzd7, then the cells were processed for endocytosis of Fzd7 by examining the internalized HA-tag antibodies. Immunofluorescence microscopy revealed that HA-Fzd7 correctly associates on the plasma membrane and can be internalized to the SNX27-containing early endosomes marked by EEA1 (Figure 2A). The endocytosis of Fzd7 is dependent of ligand stimulation, since internalization and co-localization with SNX27 were dramatically increased in the presence of Wnt3a for 1 h. Additionally, the truncated form of SNX27ΔPDZ is more cytosolic (Figure 2B), and the quantitative analysis with ImageJ software demonstrated that the co-localization of SNX27ΔPDZ with Fzd7 decreases significantly, though Fzd7 still can be internalized (Figure 2C).

**Figure 3:**
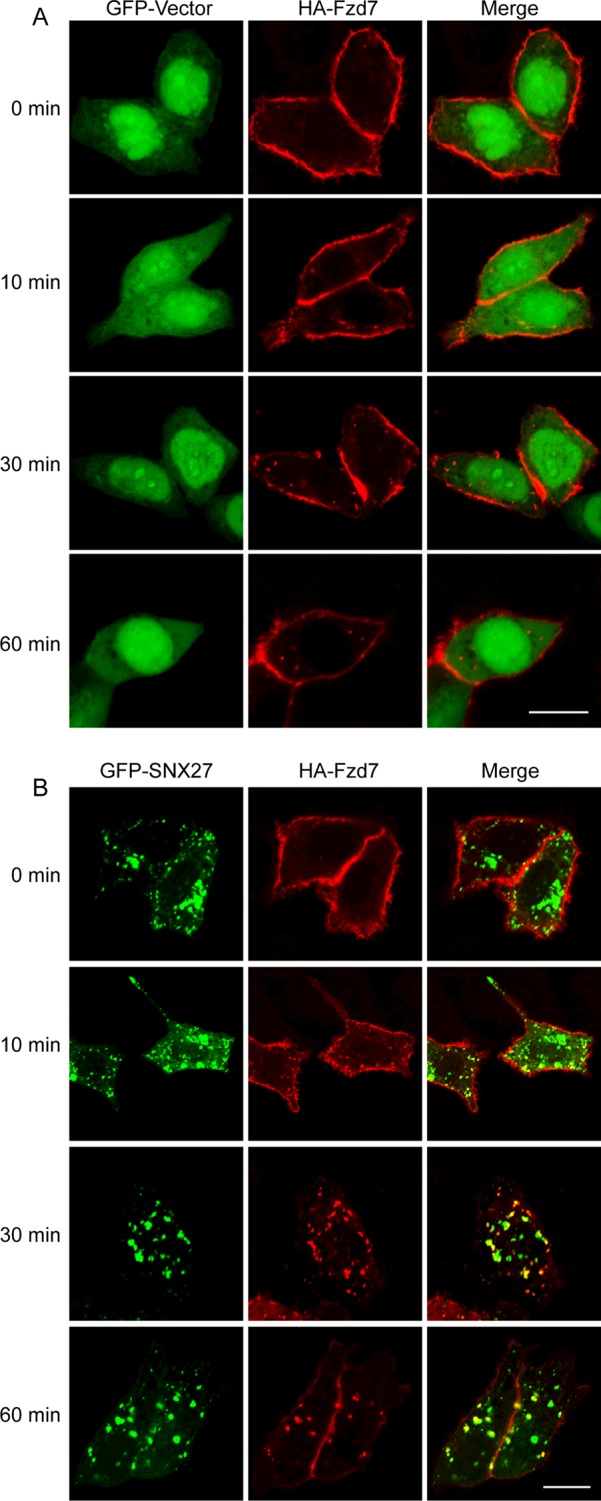
SNX27 enhances the internalization of Fzd7 (**A**) Hela cells expressing HA-Fzd7 and GFP-vector were induced for endocytosis of HA-Fzd7 with Wnt3a, and the endocytosis was terminated at different time points. (**B**) Hela cells expressing HA-Fzd7 and GFP-SNX27 were induced for endocytosis of HA-Fzd7 with Wnt3a, and the endocytosis was terminated at different time points. It was obvious that the number of HA-Fzd7-associated vesicles in cells expressing GFP-SNX27 are much more than that of cells expressing GFP-vector (A), indicating that SNX27 enhances the internalization of Fzd7. Bar=20 μm.

To further investigate the roles of SNX27 in the regulation of Fzd7 endocytosis. Hela cells were transfected with HA-Fzd7 and GFP vector or GFP-SNX27, and processed for endocytosis of HA-tag antibody by stimulated with Wnt3a for different time course. The internalization was examined by detecting the vesicle-associated HA-Fzd7. Few Fzd7-containing vesicles were observed during the time course in the cells expressing GFP vector (Figure 3A). However, the internalization was observed after stimulation with Wnt3a for 10 min, and the internalization achieves maximum after 30 min in the cells expressing GFP-SNX27 (Figure 3B), and the amount of internalized HA-Fzd7 is much more compared with that in the control cells. The results suggested SNX27 plays a role in regulating the endocytosis of Fzd7, and probably enhances the internalization of Fzd7.

### SNX27 promotes the degradation of Fzd7

**Figure 4:**
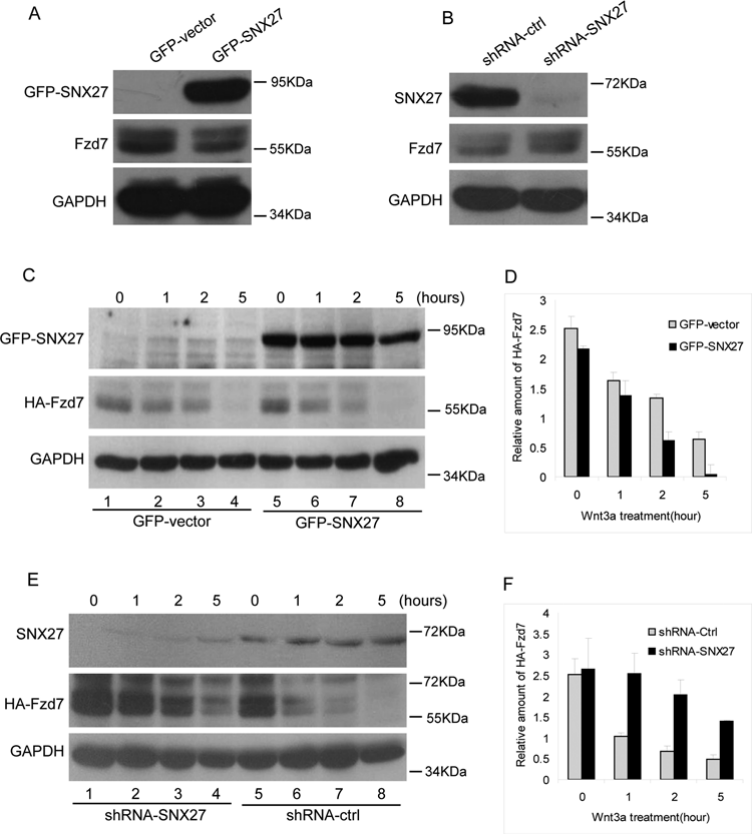
SNX27 promotes the degradation of Fzd7 (**A**) MCF7 cells stably expressing GFP or GFP-SNX27 were lysed and processed for western blot to detect Fzd7 using polyclonal antibody against Fzd7, showing less amount of Fzd7 in cells expressing GFP-SNX27 compared with that of cells expressing GFP (middle panel). (**B**) MCF7 cells stably expressing shRNA-ctrl or shRNA-SNX27 were lysed and processed for western blot to detect Fzd7 using polyclonal antibody against Fzd7, showing that Fzd7 is efficiently depleted (upper panel) and the amount of Fzd7 in SNX27 knockdown cells is much more than that of control cells (middle panel). (**C**) MCF7 cells were co-transfected with HA-Fzd7 and GFP vector or GFP-SNX27, then induced for degradation with Wnt3a for the indicated time, in the presence of cycloheximide. The cell lysates were subjected for western blot to detect HA-Fzd7 using HA-tag antibodies. The results demonstrated that over-expression of SNX27 promotes the degradation of Fzd7 (meddle panel). (**D**) Quantitative analysis of the results from three independent experiments represented in C showing that over-expression of SNX27 promotes the degradation of Fzd7. (**E**) MCF7 cells expressing shRNA-SNX27 were transfected with HA-Fzd7 then induced for degradation with Wnt3a for the indicated time. HA-Fzd7 was detected by similar approach mentioned above. The results demonstrated SNX27 knockdown significantly inhibits the degradation of Fzd7 (meddle panel). (**F**) Quantitative analysis of the results from three independent experiments represented in E showing that SNX27 knockdown inhibits the degradation of Fzd7.

The internalized surface proteins will undergo degradation or recycle back to plasma membrane. The fate of the internalized Fzd7 deserves further investigations. We examined the stability of the endogenous Fzd7 in MCF7 cells stably expressing GFP-SNX27 or pSuper.GFP-shRNA-SNX27 (with the transfection efficiency over 80%). The protein level of Fzd7 decreases upon SNX27 over-expressed (Figure 4A), but significantly increased when SNX27 is depleted by shRNA-SNX27 (Figure 4B), indicating that SNX27 influences the stability of Fzd7.

Over-expression of SNX27 reduces the cellular protein level of Fzd7, suggesting that SNX27 may promote the degradation of Fzd7. To verify this hypothesis, MCF7 cells stably expressing GFP-SNX27 or shRNA-SNX27 were transfected with HA-Fzd7, and stimulated with Wnt3a for 1, 2 or 5 h to trigger endocytosis and degradation of Fzd7. Western blot analysis demonstrated that the degradation of Fzd7 was slightly enhanced in a time course dependent manner when over-expressing SNX27 (Figures 4C and 4D). However, the degradation of Fzd7 was inhibited when SNX27 is depleted (Figures 4E and 4F). Taken together, our results suggested that SNX27 promotes the internalization and degradation of Fzd7.

### SNX27 inhibits the canonical Wnt signalling

**Figure 5:**
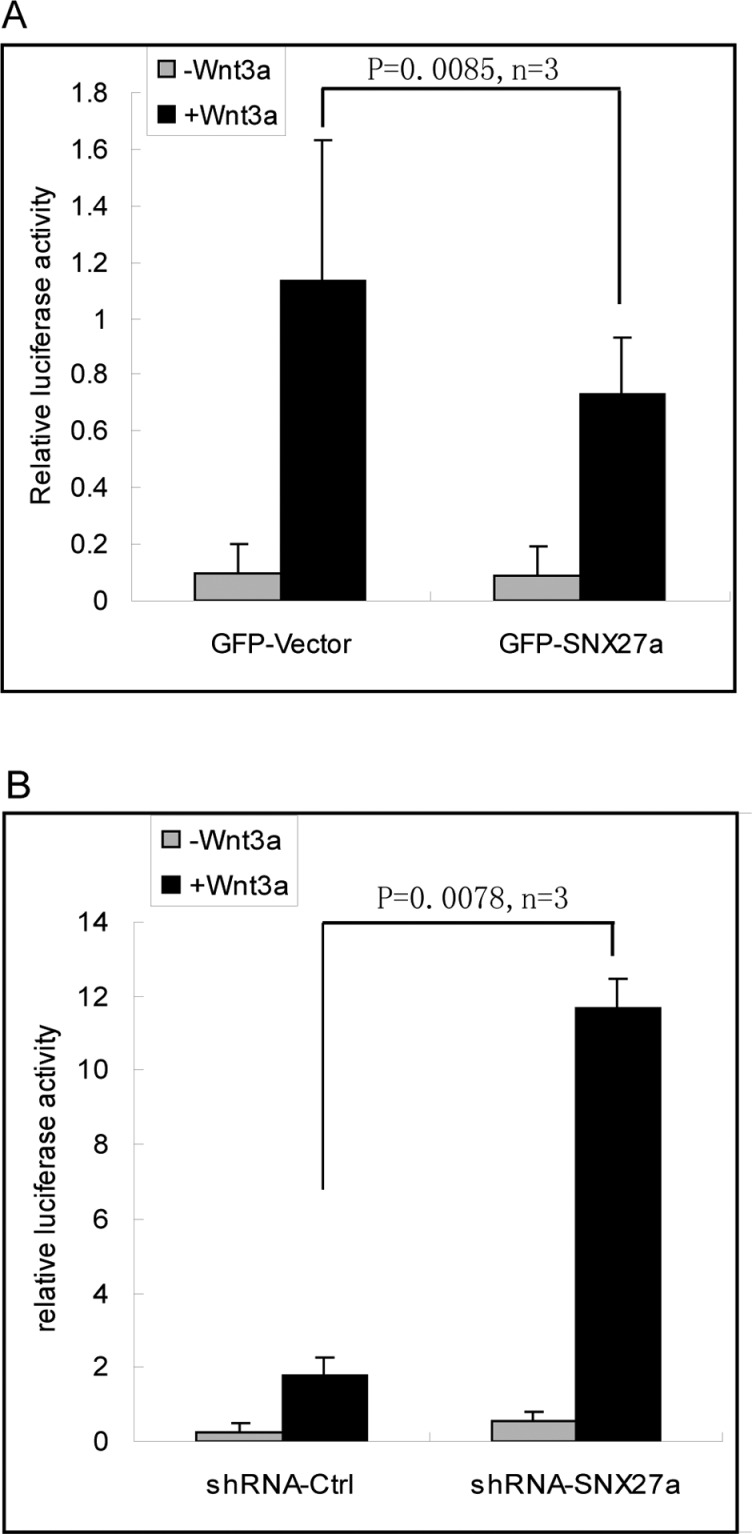
SNX27a down-regulates Wnt signalling (**A**) MCF7 cells were transfected with TOPflash and β-galactosidase plasmids, together with the indicated GFP vector or GFP-SNX27, and stimulated with 100 ng/ml Wnt3a. The cells then were processed for the detection of luciferase activity and β-galactosidase activity. The relative luciferase activity is normalized to the β-galactosidase activity. The results show that over-expression of SNX27 inhibits the Wnt regulated transcription activity. Statistical analysis was assessed by *q* test (Newman–Keuls test). (**B**) MCF7 cells expressing shRNA-ctrl or shRNA-SNX27 were transfected with TOPflash and β-galactosidase plasmids, and stimulated with 100 ng/ml Wnt3a. The transcription activity was detected similarly as mentioned above. The results demonstrate that the Wnt regulated transcription activity is greatly enhanced upon the depletion of SNX27. Statistical analysis was assessed by *q* test (Newman–Keuls test).

Wnt-Frizzled signalling will trigger the translocation of β-catenin into the nuclear to interact with TCF/LEF to activate the transcription of target genes. Since SNX27 promotes the degradation of Fzd7, we want to know whether SNX27 will influence the downstream Wnt signalling. To test the effects of SNX27 on the Wnt signalling, MCF7 cells stably expressed SNX27 or shRNA-SNX27 were transfected with pTOPflash plasmids and stimulated with Wnt3a. The transcription activity of TCF/LEF was measured by detecting the activity of luciferase normalized to the activity of β-galactosidase. The results demonstrated that over-expression of SNX27 decreases the transcription activity of TCF/LEF (Figure 5A). On the other hand, SNX27 knockdown dramatically enhances the transcription activity (Figure 5B). These results suggested that SNX27 plays an important role in regulating Wnt/β-catenin signalling.

### SNX27 inhibits cell migration

**Figure 6:**
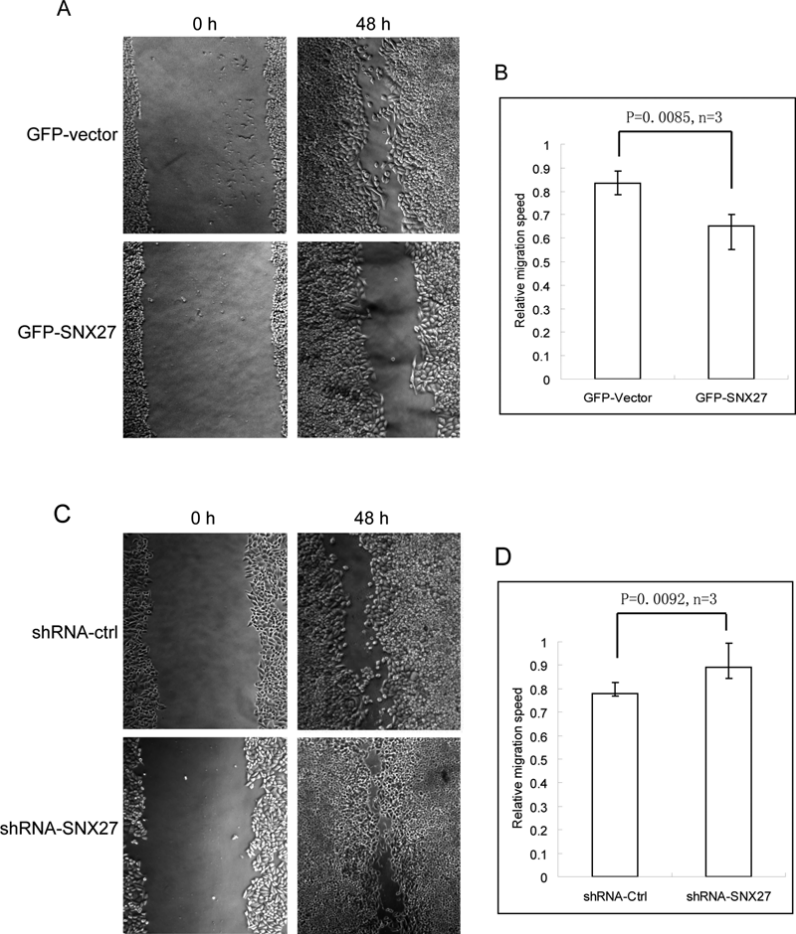
SNX27 inhibits the migration of breast cancer cells (**A**) The mono-layer of MCF7 cells stably expressing GFP-SNX27 or GFP was wounded using a yellow tip. After 48 h, the cell migration was examined under the inverted microscope. It is observed that over-expression of SNX27 inhibits the cell migration. (**B**) Quantitative analysis of the migration speed by ImageJ software from three independent experiments represented in A, statistical analysis was assessed by *t* test. (**C**) MCF7 cells stably expressing shRNA-ctrl or shRNA-SNX27 were processed for wound healing assay as mentioned above. The results revealed that SNX27 knockdown promotes the cell migration. (**D**) Quantitative analysis of the migration speed by ImageJ software from three independent experiments represented in C, statistical analysis was assessed by *t* test.

Down-regulation of Fzd7 expression inhibits invasion and metastasis of colon cancer cells [29]. SNX27 negatively influences Wnt signalling, we wondered that SNX27 plays a role in the migration of breast cancer cells. MCF cells stably expressing GFP-SNX27 or shRNA-SNX27 were processed for wound healing assay, we found that over-expression of GFP-SNX27 inhibits the migration as shown in Figures 6(A) and 6(B), whereas SNX27 knockdown promotes the migration of breast cancer cells (Figures 6C and 6D), consistent with the results of down-regulation of Wnt signalling by over-expressing SNX27 and up-regulation by SNX27 knockdown.

## DISCUSSION

SNX27 has been established to interact with multiple surface proteins including GPCR protein family through PDZ domain interaction [4–7]. Although most of Frizzled receptors contain the classic PDZ domain binding motif at the C-terminal tail, whether Frizzled receptors interact with SNX27 has not been investigated so far. In the present study, we presented data indicating that the PDZ domain binding motifs of Frizzled receptors can bind to the PDZ domain of SNX27, suggesting that Frizzled receptors act as the novel interacting partners for SNX27.

In the canonical Wnt/β-catenin signalling pathway, Wnt ligands binding to Frizzled receptors activate Dvl to inhibit GSK-3β, which stabilizes β-catenin. β-Catenin translocates to the nuclear to interact with TCF/LEF transcription factor to regulate the expression of target genes [30,31]. It has been established that Wnt/β-catenin signalling is regulated by receptor mediated endocytosis [32]. Endocytosis of Fzd is required for sustaining Wnt signalling to control planar cell polarity (PCP) [33], and inhibition of endocytosis triggers the degradation of Dvl, down-regulating Wnt/β-catenin signalling [34]. In some cases, endocytosis regulates Fzd receptors undergoing lysosomal degradation, which desensitize Wnt/β-catenin signalling [25,35]. Consistently, our results demonstrated that SNX27 promotes the internalization and degradation of Fzd7, and consequently inhibiting Wnt signalling.

So far, how Fzd receptors internalized has not been elucidated in detail. β-Arrestin regulates the endocytosis of GPCRs through clathrin-dependent manner [36]. However, β-arrestin does not interact with Fzd, though Fzd belongs to GPCR family, but interacts with Dvl [24,37], which links clathrin with Fzd [38]. In Drosophila, Fzd couples to subunit Gα_o_(G_o_) of trimeric-G protein complex to activate Wnt/PCP pathway [21]. Previous investigation indicated that G_o_ directly binds to Rab5 initiated by Fzd and activates Rab5 [39], a key regulator in mediating the internalization of receptors from plasma membrane to the early endosomes. SNX27 regulates the endocytosis and recycling of surfaces receptors in a clathrin-dependent manner [40,41]. Our results demonstrated that SNX27 is involved in the endocytosis of Fzd, which support that the internalization of Fzd requires clathrin-coated vesicular machinery. Nevertheless, our results do not exclude the possibility that SNX27 will prevent recycling of Fzd back to the plasma membrane, which deserves further investigation. Although there are not evidences showing the relationship between SNX27 and Dvl or G_o_, the early stage of endocytosis mediated by both SNX27 and/or Rab5 is crucial for Wnt/Fzd signalling.

Wnt is an important morphogen for regulating embryonic development [31]. Accumulated literature demonstrated that Wnt/β-catenin signalling is crucial for liver regeneration, metabolism and liver diseases [42–44]. It is also well established that Wnt signalling controls neural development [45,46]. And moreover, aberrant Wnt/β-catenin signalling is closely related to cancer development [47–49]. Recent works indicated that SNX27 is involved in neural disease [15]. Our works revealed SNX27 interacts with Fzd receptors and regulates Wnt/Fzd downstream signalling, indicating that SNX27 may be engaged in diverse physiological functions.

Frizzled 7 (Fzd7) is one of important member of Fzd family, previous investigations indicated that Fzd7 regulates invasion of colon cancer, emerging as novel potential target for cancer therapy [29,50,51]. Our experiments demonstrated that over-expression of SNX27 promotes the degradation of Fzd7, and inhibits Wnt/β-catenin signalling. Consistently, over-expression of SNX27 inhibits migration of breast cancer cells, whereas SNX27 knockdown enhances the cell migration. Therefore, our results exploit a novel physiological function of SNX27 in cancer development, which must be interesting for further investigations.

## Abbreviations

AMPAR: AMPA-type glutamate receptor
β2AR: β2-adrenoreceptor
Dvl: Dishevelled
GPCR: G-protein coupled receptor
mAb: monoclonal antibody
MRP4: multidrug resistance-associated protein 4
PCP: planar cell polarity
SNX: sorting nexin
